# The value of diffusion-weighted and dynamic contrast-enhanced imaging in the diagnosis of thymic epithelial tumors

**DOI:** 10.7150/ijms.76179

**Published:** 2022-09-21

**Authors:** Tran-Thi Mai Thuy, Vo Tan Duc, Tran Thanh Vy, Nguyen Hoang Nam, Nguyen Minh Duc

**Affiliations:** 1 Department of Radiology, University of Medicine and Pharmacy at Ho Chi Minh City, Ho Chi Minh City, Vietnam.; 2 Department of Radiology, University Medical Center HCMC, Ho Chi Minh City, Vietnam.; 3 Department of Cardiothoracic Surgery, University of Medicine and Pharmacy at Ho Chi Minh City, Ho Chi Minh City, Vietnam.; 4 Department of Thoracic Surgery, University Medical Center HCMC, Ho Chi Minh City, Vietnam.; 5 Department of Radiology, Pham Ngoc Thach University of Medicine, Ho Chi Minh City, Vietnam.

**Keywords:** Thymic epithelial tumor, Magnetic resonance imaging, Diffusion-weighted imaging, Dynamic contrast-enhanced imaging

## Abstract

**Background:** Thymic epithelial tumors (TETs) are clinically the most frequently encountered neoplasm of the prevascular mediastinum in adults. The role of chest magnetic resonance (MR) imaging has been increasingly stressed thanks to its excellent contrast resolution, freedom from ionizing radiation, and capability to provide additional information regarding tumors' cellular structure and vascularity.

**Methods:** This study aimed to establish the relationship between the MR findings and pathological classification of TETs, focusing on diffusion-weighted (DW) and dynamic contrast-enhanced (DCE) imaging. This retrospective cross-sectional study included 44 TET patients who underwent chest MR scanning. The tumors were classified into three groups according to the WHO classification: low-risk thymoma (LRT), high-risk thymoma (HRT), and non-thymoma (NT). Along with morphological characteristics, the apparent diffusion coefficient (ADC) value, time-intensity curve (TIC) pattern, and time to peak enhancement (TTP) of the tumors were recorded and compared between the three groups.

**Results:** A smooth contour and complete or almost complete capsule were suggestive of LRTs. The median ADC value of the 44 tumors was 0.95 × 10^-3^ mm^2^/sec. Among the three groups, LRTs had the highest ADC values, while NTs had the lowest. The differences between the ADC values of the three groups were statistically significant (p = 0.006). Using an ADC cutoff of 0.82 × 10^-3^ mm^2^/sec to differentiate between LRTs and tumors of the two remaining groups, the area under the curve was 0.775, sensitivity was 100%, specificity was 50%, and accuracy was 65.91%. The washout (type 3) TIC pattern was the most prevalent, accounting for 45.45% of the population; this pattern was also predominantly observed in LRTs (71.43%). Although the median TTP of LRTs was lower than that of HRTs or NTs, no statistically significant differences were found between the TTPs of the three groups (p = 0.170).

**Conclusions:** MR is a good imaging modality to preoperatively assess TETs. Morphological features, ADC value, TIC pattern, and TTP are helpful in preoperatively predicting TET pathology.

## Introduction

Thymic epithelial tumors (TETs) have been reported as the most common lesions of the prevascular compartment in adults. The 2021 World Health Organization (WHO) classification of thoracic tumors divides TETs into three main types, namely thymoma, thymic carcinoma (TC), and thymic neuroendocrine tumor (TNET), constituting 75-85%, 14-22%, and < 5% of cases, respectively 1.

Computed tomography (CT) is the classical imaging modality of choice to approach prevascular mediastinum lesions detected on chest radiographs. However, the role of thoracic magnetic resonance (MR) is being discussed more due to increasing evidence of its benefits of reducing unnecessary interventions and providing better tissue characterization. In addition to the advantages of not exposing patients to ionizing radiation and iodine-containing contrast agents, magnetic resonance imaging (MRI) outperforms CT in differentiating between solid and cystic lesions and distinguishing normal or hyperplastic thymus from neoplastic pathologies. Apart from conventional MR pulse sequences, diffusion-weighted (DW) imaging provides additional information on the cellular density of tumors, which is useful for detection, characterization, and therapeutic monitoring 2. The dynamic contrast-enhanced (DCE) technique helps to assess tumor perfusion, differentiate lesions, and predict the prognosis. DW- and DCE-MRI enhance the usefulness of MR, making it an indispensable element of oncological imaging.

Few studies have analyzed the characteristics of TETs with conventional MR pulse sequences as well as DW and DCE imaging. Therefore, this study was conducted with the aim of establishing the relation between the MR findings and pathological classification of TETs, with a focus on DW and DCE imaging.

## Materials and Methods

### Study design and participants

This retrospective cross-sectional study included 44 patients with TETs. The eligible patients were aged ≥ 18 years and underwent chest MR scanning at the University Medical Center (UMC), Ho Chi Minh City from September 2018 to February 2022. Patients who had received specific treatments prior to MR scanning, including chemotherapy, radiotherapy, and surgery, were excluded.

TET patients had their tumors resected or biopsied within a month of the acquisition of MR images. Experienced pathologists at the UMC examined the tumor samples and classified them into three groups: low-risk thymoma, high-risk thymoma, and non-thymoma, according to the 2021 WHO classification of thoracic tumors 1 and the simplified classification of TETs proposed by Jeong et al 3. The low-risk thymoma group comprised type A, AB, and B1 thymomas. The high-risk thymoma group included type B2 and B3 thymomas. The non-thymoma group was composed of TCs and TNETs.

### Chest MR protocol

All MR studies were performed on a 3 T unit (MAGNETOM Skyra, Siemens, Erlangen, Germany) at the UMC, Ho Chi Minh City. The conventional MR pulse sequences of the protocol included sagittal T2 HASTE (TR: 600 msec, TE: 27 msec, slice thickness: 8 mm, FOV: 350 mm × 350 mm, acquisition matrix: 256 × 320); axial T2 HASTE (TR: 1000 msec, TE: 92 msec, slice thickness: 6 mm, FOV: 260 mm × 360 mm, acquisition matrix: 240 × 320); coronal T2 HASTE (TR: 600 msec, TE: 26 msec, slice thickness: 8 mm, FOV: 400 mm × 400 mm, acquisition matrix: 256 × 320); axial T2 HASTIRM (TR: 1600 msec, TE: 86 msec, slice thickness: 6.5 mm, FOV: 300 mm × 380 mm, matrix: 260 × 320); and precontrast axial T1 VIBE DIXON (TR: 4.2 msec, TE: 1.3 msec for opposed-phase and 2.6 msec for in-phase sequences, slice thickness: 3 mm, FOV: 280 mm × 380 mm, acquisition matrix: 240 × 320).

DW images were obtained by using an echo-planar sequence (TR: 6500 msec, TE: 72 msec, slice thickness: 6 mm, FOV: 320 mm × 400 mm, acquisition matrix: 120 × 150) with b values of 0, 500, and 2000 sec/mm^2^. ADC maps were automatically constructed by the machine's software and displayed simultaneously after DW images were acquired.

DCE sequencing was performed using axial T1 VIBE DIXON after 30 sec, 60 sec, 90 sec, 2 min, 3 min, 4 min, and 5 min after bolus administration of gadoterate meglumine (Dotarem, Guerbet, France; 0.1 mmol/kg) at a rate of 1-2 ml/sec. The parameters of the postcontrast sequences were similar to those of the precontrast axial T1 VIBE DIXON.

### Data sampling

**Figure 1:**
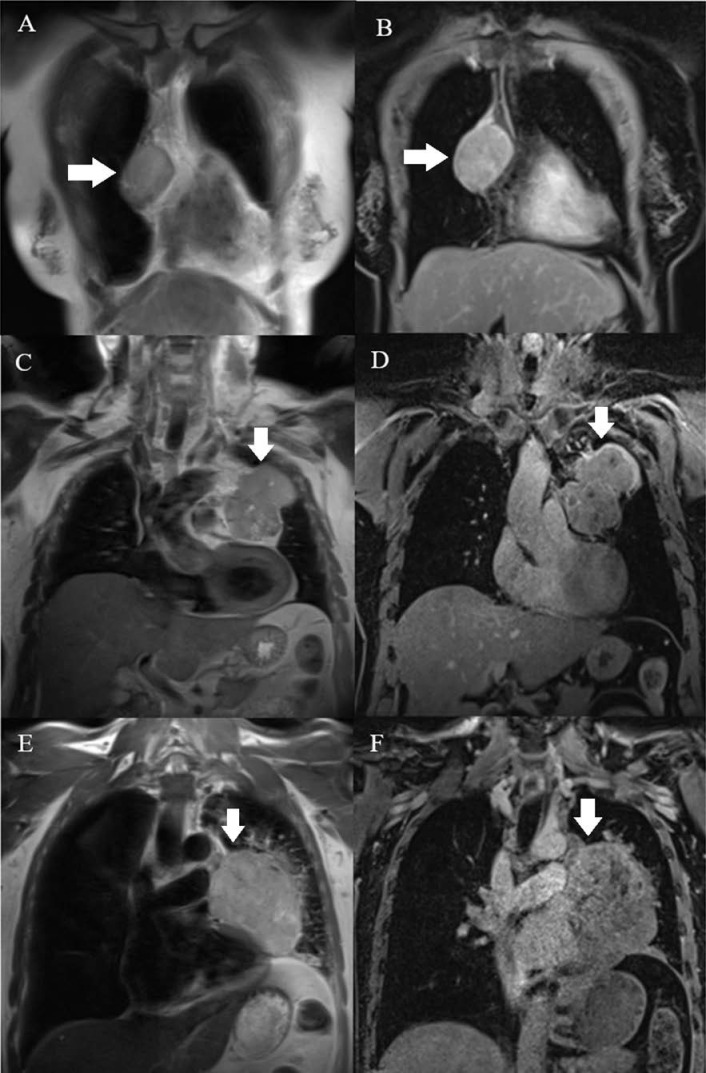
Capsules (arrow) were assessed on T2W (A, C, E) and postcontrast T1W (B, D, F) images. **A, B:** A type AB thymoma with an almost complete capsule. **C, D:** A type B2 thymoma with a partial capsule. **E, F:** A TC without a visible capsule.

MR images were evaluated using a RadiAnt DICOM Viewer (Medixant, RadiAnt DICOM Viewer Version 2021.1. Jun 27, 2021, URL: https://www.radiantviewer.com) by a senior radiologist who was blinded to the final histological diagnosis. The collected MRI characteristics included the longest diameter, border, cystic or necrotic or hemorrhagic component, capsule, septum, pleural implant, pleural or pericardial effusion, and mediastinal lymphadenopathy. The border was assessed as being either smooth or lobulated. Tumors with a smooth contour were typically round or ovoid in shape. A lobulated border exhibited one or more lobulations with the presence of notches between tumor lobules. Cystic, necrotic, or hemorrhagic components were presumed to be areas of non-enhancing fluid that could be simple or complicated. Simple fluid or serous fluid had the same signal intensity as cerebrospinal fluid on all pulse sequences, which was homogenously T1 hypointense and T2 hyperintense and showed no restricted diffusion. On the other hand, complicated fluid, such as proteinaceous fluid or blood, had variable signal features on T1-weighted (T1W), T2-weighted (T2W), and DW images. Tumor capsules were defined as thin rims located in the periphery of the tumor and having the characteristic signal intensity of fibrous tissue, i.e., T1 hypointense, T2 hypointense, and better visualized in the late phases of DCE MRI. Capsule integrity was defined as complete or almost complete if the capsules were visible for more than two-thirds of the perimeter of the tumor, partial if they were visible for less than two-thirds of the perimeter of the tumor, or absent (Fig. 1). Tumor septa were defined as thin linear, curved, or reticular structures dividing the tumor into lobules and often having similar signal intensity characteristics to tumor capsules, i.e., hypointense on T1W and T2W images. Mediastinal lymphadenopathy was defined as the presence of mediastinal lymph nodes with a short axis diameter of ≥ 10 mm.

The ADC values ​​of the solid component were acquired by manually placing regions of interest (ROI) on the ADC map at three different sites of solid tissue having the lowest signal intensities with a minimal ROI area of 0.5 cm^2^. When placing the ROI, the interferences between the tumor and surrounding lung tissue or major blood vessels were avoided. Areas of necrotic, cystic, or hemorrhagic components were also excluded. The mean ADC value in the ROI was recorded and the average of three ADC values was then obtained. For DCE-MRI, two variables, namely the time-intensity curve (TIC) pattern and time to peak enhancement (TTP), were collected by constructing TICs of three sites of solid tissue that were previously placed on the ADC map by ROI using the TIC-generating tool within the RadiAnt DICOM Viewer and then choosing the earliest TIC-reaching peak enhancement as representative of the tumor's solid tissue. TIC pattern was classified as type 1 (gradual), type 2 (plateau), or type 3 (washout) according to the semi-quantitative method proposed by Yabuuchi et al. 4,5. Two parameters defining the pattern of TIC, TTP, and washout ratio (WR) could be detected and calculated based on the chosen TIC. TTP was presumed to be the time at which ROI-placed solid tissue reached the highest signal intensity. If the TIC continued upward at the end of the DCE phases, TTP was counted as 5 min. WR was calculated using the equation WR = (SI_max_ - SI_5 min_)/ (SI_max_ - SI_pre_), where SI_max_: signal intensity at peak enhancement, SI_5 min_: signal intensity in the final phase of DCE MRI, and SI_pre_: precontrast signal intensity. Type 1 TIC had TTP > 120 sec. Type 2 TIC had TTP ≤ 120 sec and WR < 30%. Type 3 TIC had TTP ≤ 120 sec and WR ≥ 30%.

### Statistical methods

Data were presented as frequency and percentage for qualitative variables. For non-normally distributed quantitative variables, data were presented as median and interquartile range. The Chi-squared test and Fisher's exact test were used to compare proportions between the three groups (border, cystic/necrotic/hemorrhagic component, capsule, septum, pleural or pericardial effusion, pleural metastases, and mediastinal lymphadenopathy). The Kruskal-Wallis test was used to compare the ADC values and TTPs of the three groups. The Mann-Whitney test was used to compare the longest diameter and TTP between thymoma and non-thymoma. A receiver operating characteristic (ROC) curve was drawn to detect the ADC value cutoff to differentiate low-risk thymoma from high-risk thymoma and TC, with the calculation of sensitivity, specificity, and accuracy. Data were analyzed with STATA version 15.1 (StataCorp. 2017. Stata Statistical Software: Release 15, StataCorp LLC, College Station, TX, USA). P values ≤ 0.05 were considered to indicate statistical significance.

### Ethical considerations

The present study was approved by the Ethics Committee of the University of Medicine and Pharmacy in Ho Chi Minh City and the UMC (IRB-VN01002/IORG0008603/FWA00023448, dated 17 December 2021). Informed consent was obtained from all individual patients included in the study.Our study was performed in compliance with the principles outlined in the Declaration of Helsinki.

## Results

### Pathological characteristics

Our study included 44 patients (17 males and 27 females), each having only one tumor. There were 14 low-risk thymomas, 18 high-risk thymomas, nine TCs, and three TNETs in the cohort. Type AB and type B2 thymomas were the two most common pathological findings, constituting 27.27% and 25.00% of the total (Table 1).

### Morphological MR features

Morphological MR data are summarized in Table 2. In the current study, the longest diameter of the TETs ranged from 18-186 mm, with a median of 68.5 mm. The median longest diameter was 51 mm for low-risk thymomas, 55 mm for high-risk thymomas, and 92.5 mm for non-thymomas. Thymomas, including the high-risk and low-risk variants, had a median longest diameter of 54.5 mm. While no statistically significant differences were found between the longest diameter of low-risk thymomas and high-risk thymomas (p = 0.436), non-thymomas were significantly larger than thymomas (p = 0.001).

64.29% of low-risk thymomas had smooth contours, whereas lobulated contours were the predominant finding in high-risk thymomas (94.44%) and non-thymomas (100%). Cystic, necrotic, or hemorrhagic components were identified in 21 of the 44 TETs included (47.73%). There were no significant differences in the frequency of cystic, necrotic, or hemorrhagic components between the three groups (p = 0.084).

Regarding the presence and integrity of fibrous capsules, complete or almost complete capsules were most common in low-risk thymomas (8/14, 57.14%). In contrast, partial capsules were primarily found in high-risk thymomas and non-thymomas, comprising 83.33% and 75%. Among the 44 tumors, we could not identify a capsule in one low-risk thymoma and three non-thymomas. Pleural or pericardial effusion was present in one of 14 low-risk thymomas (7.14%) and three of 18 high-risk thymomas (16.67%), while this finding was predominant in non-thymomas (8/12, 66.67%). Of all patients, only two cases of high-risk thymomas presented with pleural implants. Mediastinal lymphadenopathy was primarily observed in non-thymoma cases (5/12, 41.67%).

### DW- and DCE-MR features

**Figure 3:**
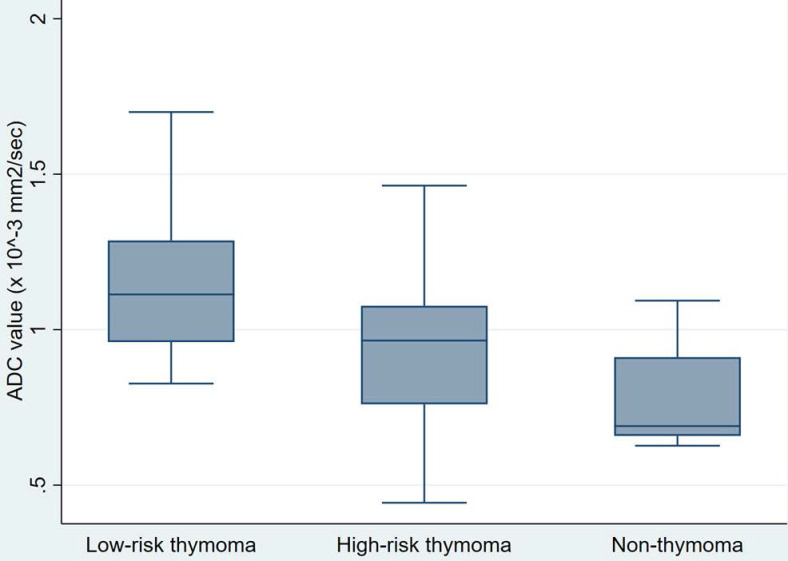
Box and whisker plots showing the comparison of the ADC values of three groups of TETs. Despite the overlap between the three distinct groups, the ADCs of low-risk thymomas were significantly higher than those of high-risk thymomas and TCs.

**Figure 4:**
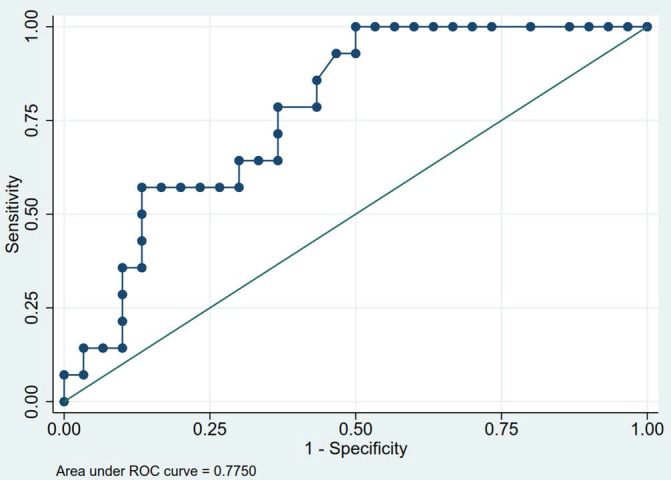
The ROC curve of ADC values to differentiate low-risk thymomas from tumors of the two remaining groups. The optimal ADC cutoff was 0.82 × 10^-3^ mm^2^/sec and AUC was 0.775.

Table 3 exhibits the DW- and DCE-MR characteristics of the patients. The median ADC value of the 44 tumors included in this study was 0.95 × 10^-3^ mm^2^/sec. Low-risk thymomas had the highest ADC value of 1.11 × 10^-3^ mm^2^/sec, while high-risk thymomas and non-thymomas had values of 0.97 × 10^-3^ mm^2^/sec and 0.69 × 10^-3^ mm^2^/sec, respectively (Fig. 3). The differences between the ADC values of low-risk thymomas, high-risk thymomas, and non-thymomas were statistically significant (p = 0.006). The area under the ROC curve (AUC) of the ADC value in differentiating between low-risk thymomas and tumors of the two remaining groups was 0.775 (Fig. 4). With an optimal cutoff point of 0.82 × 10^-3^ mm^2^/sec (Youden Index J = 0.500), sensitivity was 100%, specificity was 50%, and accuracy was 65.91% (Table 4).

The washout pattern (type 3) of TIC was the most common among the TETs, constituting 45.45% of all cases. This pattern was also predominantly observed in low-risk thymomas (10/14, 71.43%). On the other hand, the plateau pattern (type 2) was predominant in the two remaining groups, observed in 44.44% of high-risk thymomas and 41.67% of non-thymomas. Low-risk thymomas had the lowest median TTP (60 sec) of the three groups. High-risk thymomas and non-thymomas reached peak enhancement later, with a median TTP of 151 sec and 135 sec, respectively. Thirty-two thymomas had a median TTP of 120 sec (interquartile range: 45-180). The differences between the TTPs of low-risk thymomas, high-risk thymomas, and non-thymomas were insignificant (p = 0.170). Additionally, no significant differences were found between the TTPs of thymomas and non-thymomas (p = 0.522).

## Discussion

The oncological significance of the WHO histological classification of TETs is well recognized in clinical practice and research 1. Type A, AB, and B1 thymomas are more likely to be completely resectable than type B2 and B3 thymomas and non-thymomas. Moreover, adjuvant or neoadjuvant therapy may be needed for high-risk thymomas and non-thymomas, while low-risk thymomas may only require thymectomy. Preoperatively predicting TET histopathology plays a pivotal role in planning treatment strategies and prognostication 3. As few studies on TNET have been published, we decided to include TNETs in this study under the categorization of non-thymoma along with TCs because of their similar aggressive behavior, poor prognosis, and imaging features.

**Figure 5:**
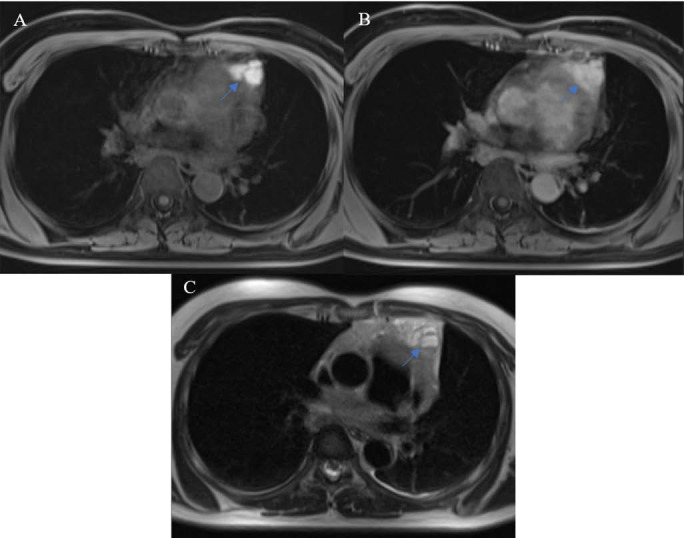
A thymic neuroendocrine tumor having a characteristic reticular septum within a hemorrhagic component that had high T1 signal intensity and mixed T2 signal intensity with fluid-fluid levels. **A:** precontrast, fat-saturated T1W image. **B:** postcontrast, fat-saturated T1W image. **C:** T2W image.

In our study, the longest diameter of non-thymomas was significantly larger than that of thymomas. This finding is comparable to the studies of Tomiyama et al. 6 and Jung et al. 7. Jeong et al. 3 reported that smooth contours were predominantly observed in low-risk thymomas, while lobulated contours were more common in high-risk thymomas and TCs. This is in accordance with our observation of 44 TETs and can be explained by the speculation that more aggressive tumors tend to present with lobulated contours due to their rapid growth. There were no significant differences regarding the presence of fluid components between the three groups. Nonetheless, the signal features of fluid components may play a role in characterizing TETs. According to a study published in the British Institute of Radiology 8, a reticular septum within a hemorrhagic component may be characteristic of TNET. In our study, all TNETs demonstrated the presence of fluid components with signal features suggestive of blood products (Fig. 5); gross and microscopic examination confirmed the presence of intratumoral hemorrhage.

Pathologically, the fibrous capsule and septum are two components known to help differentiate between thymomas and lymphomas, which are the most commonly encountered entities in the prevascular mediastinum of adults 9. Several reports showed that MR, owing to its excellent contrast resolution, was better than CT for visualizing the capsules and septa of mediastinal tumors 10,11. Comprising primarily fibrous tissue, the capsules and septa of TETs have characteristic MR signal features, i.e., T1 hypointense, T2 hypointense, and better visualized in the late phases of DCE-MRI. In the current study, the appearance of a complete or almost complete capsule on the MRI was suggestive of low-risk thymomas, while high-risk thymomas and non-thymomas predominantly exhibited partial capsules. This is in accordance with a study by Sadohara et al 11 and their pathological observation that more aggressive TETs often demonstrated capsular invasion at presentation. According to the Masaoka-Koga staging system 12, the sole presence of pleural implants categorizes a TET as stage IVa. When the primary tumor is off-midline, ipsilateral pleural metastases are found. They must be separate from the primary tumor and tend to share signal characteristics with it. In a study investigating nine thymomas and 18 TCs 13, pleural dissemination was found in one thymoma and six TCs. The difference in the frequency of pleural dissemination between the two groups was not significant. In our study, pleural lesions were only detected in two high-risk thymomas. Although considered unspecific, pleural effusion was observed primarily in more aggressive tumors in several TET imaging studies 6. In our work, pleural effusion was observed in only one patient with type AB thymoma, while more than 50% of patients with non-thymomas presented with an appreciable amount of pleural fluid on MR images. The utility of MR in detecting metastatic lymph nodes is questionable, even with the aid of advanced techniques including DWI. In the current study, lymph nodes were evaluated solely based on short axis diameter, although signal and other morphological characteristics may play a specific role in evaluation. Mediastinal lymphadenopathy was seen in two cases (one low-risk and one high-risk case), which contrasts with the considerable frequency of this finding in the non-thymoma group (5/12, 41.67%). This finding is compatible with the tendency of thymomas to invade locally rather than spreading lymphatically and hematogenously 14.

**Figure 6:**
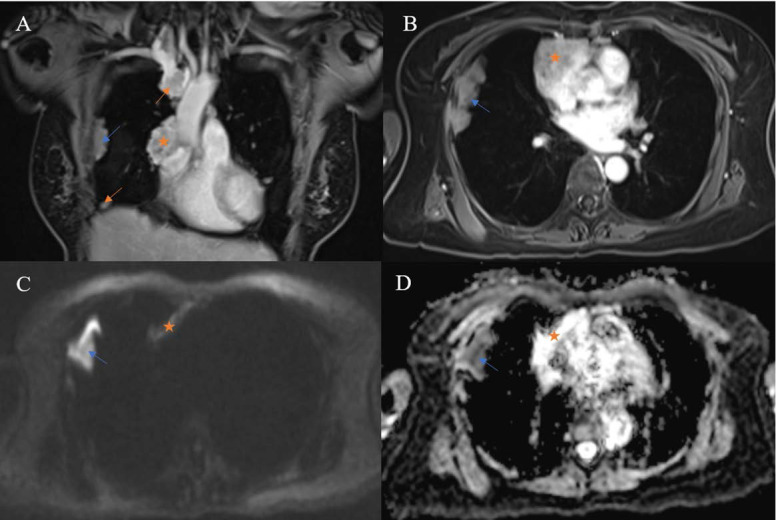
Multiple pleural metastases (arrows) were noted in a 60-year-old woman with type B2 thymoma. Pleural lesions were ipsilateral to the primary tumor (star). A pleural plaque (blue arrow) had lower ADC than the primary tumor (0.5 × 10^-3^ and 1.11 × 10^-3^ mm^2^/sec, respectively).

DW-MRI has been used to differentiate between malignant and benign tumors in various body regions. The advent of the echo-planar technique has made DW-MRI of the thorax possible owing to rapid image acquisition that minimizes the effects of physiological motion from respiration and cardiac movements 15. The pathological differences between benign lesions and malignancies explain the discrepancy in their ADC values. The characteristics of malignant tumors, including hypercellularity, enlarged nuclei, hyperchromatism, and angulation of the nuclear contour, contribute to reducing the extracellular matrix and the diffusion space of water protons, which in turn causes a decrease in the ADC value 16. Acquiring DW images with b values of 0, 400, and 800 in a 1.5 T unit, Razek et al. 17 found that the ADC values of low-risk thymoma, high-risk thymoma, and TC were 1.30 × 10^-3^ mm^2^/sec, 1.16 × 10^-3^ mm^2^/sec, and 1.18 × 10^-3^ mm^2^/sec, respectively. In the same study, the AUC was 0.804 when using an ADC cutoff of 1.25 × 10^-3^ mm^2^/sec to differentiate low-risk thymomas from high-risk thymomas and thymic carcinomas. A recent study by Shen et al. 18 reported that the mean ADC values of low-risk thymoma, high-risk thymoma, and TC were 1.279 × 10^-3^ mm^2^/sec, 0.978 × 10^-3^ mm^2^/sec, and 0.661 × 10^-3^ mm^2^/sec, respectively. The optimal ADC cutoff to distinguish low-risk thymomas from high-risk thymomas and TCs in the research of Shen et al. 18 was 1.193 × 10^-3^ mm^2^/sec, which was also lower than the value reported by Razek et al. 17. The ADC values of the TETs in our study were lower compared with the results of both Razek et al. 17 and Shen et al. 18. The difference may be due to variations in the chosen DW-MRI parameters, specifically b values. Technically, the ADC value decreases when the b values increase 19. Our study employed b values of 0, 500, and 2000, while Razek et al. 17 and Shen et al. 18 used values of 0, 400, and 800 sec/mm^2^ and 0 and 1000 sec/mm^2^, respectively. Additionally, due to the rarity of TETs, the sample sizes of all three studies were relatively small, making them prone to selection bias. It should be noted that while TNETs were included in our study, the other two studies did not include such cases. We observed a type B3 thymoma having an ADC value of 1.56 × 10^-3^ mm^2^/sec and a TC having an ADC value of 1.37 × 10^-3^ mm^2^/sec. Both values were considerably higher than the median ADCs of their groups, which may be due to the extensive micronecrosis observed pathologically. Unexpectedly, pleural lesions in a case of type B2 thymoma had a lower ADC than the primary tumor (Fig. 6).

**Figure 7:**
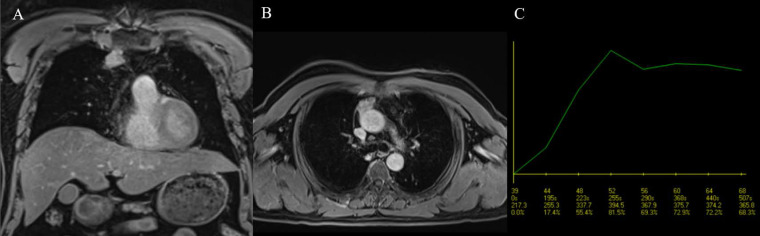
A type B2 thymoma having its TIC classified as type 2 and a TTP of 90 sec. **A:** Coronal, postcontrast, fat-saturated T1W image. **B:** Axial, postcontrast, fat-saturated T1W image. **C:** Plotted TIC.

The usefulness of the TIC pattern and semi-quantitative parameters extracted from DCE-MRI data, particularly TTP, in the evaluation of prevascular mediastinal tumors has been suggested by previous research. According to a study by Yabuuchi et al. 5 characterizing prevascular mediastinal tumors in adults using MR and positron-emission tomography/computed tomography (PET/CT), TETs had a primary TIC pattern of washout (38/71, 53.5%), and this TIC type was seen exclusively in TETs. The authors hypothesized that this observation could be due to TETs' high cellularity and scarcity of stroma. They also reported a predominance of the washout pattern in the low-risk thymoma group (54.9%) compared to the high-risk thymoma (47.06%) and TC groups (15.38%). In our study, the washout pattern of TIC was the most common among TETs (45.45%), and this pattern was predominant in low-risk thymomas, similar to the results of Yabuuchi et al. 5. Some studies reported that the TTP of thymomas was lower than that of TCs or TNETs, potentially due to thymomas' higher microvessel density 20. Thymomas in a study by Sakai et al. 20 had a TTP of 1.5 ± 0.9 min, while non-thymomas (including TCs and TNETs) in the same study showed a significantly higher value of 3.2 ± 1.2 min. In a study conducted by Lin et al. 21, 31 thymoma patients exhibited significantly lower TTPs than 14 patients with TCs (89 sec and 172 sec, respectively). The authors also found that TTP was among the top three DCE-MRI parameters for distinguishing between thymomas and TCs, with an AUC of 0.779 based on univariate analysis 21. In our study, the TTP of thymomas had a median value of 120 sec (interquartile range: 45-180), which is comparable to the work of Sakai et al. 20 and Lin et al. 21 (Fig. 7); however, the TTP of high-risk thymomas in our study was higher than that of non-thymomas, which is in contrast to their observations. We also noticed that although the median TTP of low-risk thymomas was lower than that of high-risk thymomas or non-thymomas, no statistically significant differences were found between the TTPs of the three groups. Two out of the three included TNETs had TTPs of 30 sec, which was lower than the median TTP of low-risk thymomas (60 sec) and against the previously mentioned expectation of thymomas reaching peak enhancement sooner than more aggressive tumors. Nevertheless, the number of TNETs in this study was too low to make any speculations. Interestingly, other research on utilizing the TTP of DCE-MRI to differentiate between benign and malignant lesions of the breast and ovary showed the opposite result 22,23: benign lesions had a higher TTP compared to cancer of these organs. Thyroid lesions exhibited concordant findings with TET: the washout pattern of TIC and a low TTP indicated a benign lesion rather than thyroid carcinoma 24.

This study had several limitations. First, the study was limited by a small sample size. Second, ADC value, TIC pattern, and TTP were based on manually determined ROIs such that the results were susceptible to errors caused by choosing unrepresentative regions of tumor solid tissue. Third, DCE-MRI data were assessed by semi-quantitative analysis, which is strongly dependent on the hardware, parameters of MRI pulse sequences, and dose of contrast agents, leading to difficulties when comparing results between institutions. Further studies with larger sample scale with prospective and multicenter design are essential to validate our findings. In addition, the combination the conventional MRI with diffusion-weighted and dynamic contrast-enhanced imaging should be considered in future research to enhance the diagnostic performance.

## Conclusion

Though CT has been the modality of choice to evaluate TETs thus far, MR has superior contrast resolution and can provide additional information regarding tissue cellularity and vascularity. Some morphological features, including lobulated contours, presence of pleural or pericardial effusion, and presence of pleural implants, favor the diagnosis of a more aggressive tumor over a low-risk thymoma. The ADC value is a useful tool to differentiate low-risk thymomas from high-risk thymomas and non-thymomas; almost perfect sensitivity was achieved when using a cutoff of 0.82 × 10^-3^ mm^2^/sec. High-risk thymomas and non-thymomas had higher median TTPs compared to low-risk thymomas. Nevertheless, studies with larger populations should be conducted to better demonstrate the correlation between the semi-quantitative parameters of DCE-MRI and the WHO pathological classification of TETs.

## Figures and Tables

**Figure 2:**
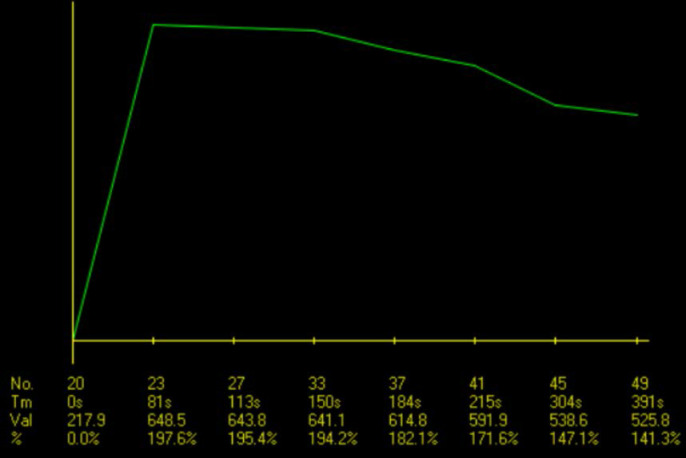
The type 3 TIC of a type AB thymoma, with a TTP of 30 sec.

**Table 1:** Pathological features

Pathology	Frequency	Percentage
*Low-risk thymoma*	***14***	
Type A thymoma	1	2.27%
Type AB thymoma	12	27.27%
Type B1 thymoma	1	2.27%
*High-risk thymoma*	***18***	
Type B2 thymoma	11	25.00%
Type B3 thymoma	7	15.91%
*Non-thymoma*	***12***	
Thymic carcinoma (TC)	9	20.45%
Thymic neuroendocrine tumor (TNET)	3	6.82%

**Table 2:** Morphological MR findings

Variable	Low-risk thymoma (n = 14)	High-risk thymoma (n = 18)	Non-thymoma (n = 12)	P value
Longest diameter (mm)^a^	51 (38-72)	55 (45-76)	92.5 (78-107.5)	0.003^c^
**Border^b^**				0.000^d^
Smooth	9 (64.29%)	1 (5.56%)	0 (0%)	
Lobulated	5 (35.71%)	17 (94.44%)	12 (100%)	
**Cystic, necrotic, or hemorrhagic component^b^**				0.084^d^
Present	5 (35.71%)	7 (38.89%)	9 (75%)	
Absent	9 (64.29%)	11 (61.11%)	3 (25%)	
**Capsule^b^**				0.002^d^
Complete or almost complete	8 (57.14%)	3 (16.67%)	0 (0%)	
Partial	5 (35.71%)	15 (83.33%)	9 (75%)	
None	1 (7.14%)	0 (0%)	3 (25%)	
**Septum^b^**				0.024^d^
Yes	2 (14.29%)	11 (61.11%)	4 (33.33%)	
No	12 (85.71%)	7 (38.89%)	8 (66.67%)	
**Pleural or pericardial effusion^b^**				0.001^d^
Yes	1 (7.14%)	3 (16.67%)	8 (66.67%)	
No	13 (92.86%)	15 (83.33%)	4 (33.33%)	
**Pleural metastases^b^**				0.220^d^
Yes	0 (0%)	2 (11%)	0 (0%)	
No	14 (100%)	16 (88.89%)	12 (100%)	
**Mediastinal lymphadenopathy^b^**				0.017^d^
Yes	1 (7.14%)	1 (5.56%)	5 (41.67%)	
No	13 (92.86%)	17 (94.44)	7 (58.33%)	

^a^ Data are presented as median (inter-quartiles). ^b^ Data are presented as frequency (percentage). ^c^ P value was calculated using the Kruskal-Wallis test. ^d^ P values were calculated using the Fisher's exact test.

**Table 3:** ADC value, TIC type, and TTP

Variable	Low-risk thymoma (n = 14)	High-risk thymoma (n = 18)	Non-thymoma (n = 12)	P value
ADC value (× 10^-3^ mm^2^/sec)^a^	1.11 (0.96-1.29)	0.97 (0.76-1.08)	0.69 (0.66-0.91)	0.006^c^
**TIC type^b^**				
Gradual	1 (7.14%)	4 (22.22%)	3 (25%)	0.220^d^
Plateau	3 (21.43%)	8 (44.44%)	5 (41.67%)	
Washout	10 (71.43%)	6 (33.33%)	4 (33.33%)	
TTP (sec)^a^	60 (30-180)	151 (90-180)	135 (60-300)	0.170^c^

^a^ Data are presented as median (inter-quartiles). ^b^ Data are presented as frequency (percentage). ^c^ P values were calculated using the Kruskal-Wallis test. ^d^ P values were calculated using the Fisher's exact test.

**Table 4:** Diagnostic performance of ADC values in differentiating low-risk thymomas from high-risk thymomas and non-thymomas

	AUC	Sensitivity	Specificity	Accuracy
ADC value > 0.82(× 10^-3^ mm^2^/sec)	0.775	100%	50%	65.91%
